# Large Number Discrimination in Newborn Fish

**DOI:** 10.1371/journal.pone.0062466

**Published:** 2013-04-23

**Authors:** Laura Piffer, Maria Elena Miletto Petrazzini, Christian Agrillo

**Affiliations:** Department of General Psychology, University of Padova, Padova, Italy; Monash University, Australia

## Abstract

Quantitative abilities have been reported in a wide range of species, including fish. Recent studies have shown that adult guppies (*Poecilia reticulata*) can spontaneously select the larger number of conspecifics. In particular the evidence collected in literature suggest the existence of two distinct systems of number representation: a precise system up to 4 units, and an approximate system for larger numbers. Spontaneous numerical abilities, however, seem to be limited to 4 units at birth and it is currently unclear whether or not the large number system is absent during the first days of life. In the present study, we investigated whether newborn guppies can be trained to discriminate between large quantities. Subjects were required to discriminate between groups of dots with a 0.50 ratio (e.g., 7 vs. 14) in order to obtain a food reward. To dissociate the roles of number and continuous quantities that co-vary with numerical information (such as cumulative surface area, space and density), three different experiments were set up: in Exp. 1 number and continuous quantities were simultaneously available. In Exp. 2 we controlled for continuous quantities and only numerical information was available; in Exp. 3 numerical information was made irrelevant and only continuous quantities were available. Subjects successfully solved the tasks in Exp. 1 and 2, providing the first evidence of large number discrimination in newborn fish. No discrimination was found in experiment 3, meaning that number acuity is better than spatial acuity. A comparison with the onset of numerical abilities observed in shoal-choice tests suggests that training procedures can promote the development of numerical abilities in guppies.

## Introduction

The study of quantitative ability in non-human animals represents one of the main topics of research in animal cognition [1], [2], [3]. There are indeed many real-life situations in which using quantitative abilities can be useful, and there is no reason to believe that selective pressures in favour of the ability to quantify different magnitudes should have acted only on hominids. Quantitative abilities can permit animals to optimize foraging, enabling them to rapidly select the largest of two available sources of food [4], [5], [6]; this ability also represents a powerful tool for anti-predator defence, letting animals join the largest available group of conspecifics, reducing the probability of being spotted by predators [7], [8]. Quantitative abilities also are useful in social interactions; chimpanzees, lionesses and hyenas are more likely to attack other groups of conspecifics when they perceive themselves as being part of a larger group [9], [10], [11].

However, discrete numerical information may co-vary with other continuous attributes of the stimuli, such as cumulative surface area, density or the overall space occupied by the sets, and animals can potentially use the relative magnitude of continuous quantities to assess the numerical size of a group [12], [13]. Despite the large amount of studies conducted over the past decade, the relative salience of numerical information to continuous quantities is unclear: While some studies reported a spontaneous use of numerical information [14], [15], others reported a preferential or exclusive use of continuous quantities [16], [17], [18], suggesting that numerical information is more cognitively demanding than continuous quantities and raising the possibility that several species might use numbers only as a last resort strategy when no other continuous information is available [19].

Recently, quantitative abilities have been investigated in fish [8], [20]. Mosquitofish, for example, proved to be able to process both numerical and continuous information in free choice tests and after extensive training [15], [21]. Using adult guppies’ spontaneous preference to go to the largest shoal when placed in an unfamiliar environment, Agrillo and colleagues [7] showed that adult guppies are particularly accurate in the range 1–4, discriminating 1 vs. 4 (ratio 0.25), 1 vs. 3 (0.33), 1 vs. 2 (0.50), 2 vs. 3 (0.67) and 3 vs. 4 (0.75) with the same accuracy, thus showing no influence of numerical ratio in small quantities. In contrast, guppies’ ability with larger quantities (>4) of the same ratios showed ratio dependence, with accuracy decreasing as numerical ratios increased. These data suggest the potential use of different quantificational systems: a precise system for up to 4 units and an approximate one for larger numbers, as advanced in mammals [22], [23], [24], [25] and birds [26].

The suggestion of different abilities in handling small and large quantities is also supported by a recent study that investigated the ontogeny and development of quantitative ability in newborn guppies. One-day-old guppies proved able to discriminate between shoals when the choice involved small quantities (1 vs. 2, 2 vs. 3, 3 vs. 4) but not when larger groups were presented (4 vs. 5, 4 vs. 8 or even 4 vs. 12). Such abilities seem to emerge only later, between 20 and 40 days of age [27]. This study suggests that, for guppies, the ability to discriminate among small quantities is innate and displayed at birth, at least in the social domain, while the ability to discriminate among large quantities is not present at birth and emerges only later. The apparent inability to distinguish even a 0.33 ratio is surprising and raises the intriguing question as to whether the cognitive mechanisms underlying the capacity for large quantity discrimination could be promoted through a training program.

The aim of the present study is to investigate whether trained newborn guppies can discriminate among large quantities. To this purpose we used the method recently set up by Miletto Petrazzini et al. [28] which diverges from a standard operant conditioning procedure. In this method the fish are housed in rectangular tanks. At intervals, two stimuli (dots in different quantities) are introduced at opposite ends of the tank, and food is delivered near the largest quantity. Discrimination is inferred from the proportion of time spent near the reinforced stimulus during probe trials at the end of training, instead of from individual learning criterion. The method proved to be very rapid and hence allows to study age–specific cognitive abilities even in a rapidly growing species, such as guppies [28]. To disentangle the roles of numerical and continuous information, fish were observed in three different quantity conditions: a) number and continuous quantities both could be used, b) only number could be used, and c) only continuous quantities could be used.

## Materials and Methods

### Ethics Statement

The Experiments comply with all laws of the country (Italy) in which it was performed (D.M. 116192) and was approved by ‘Ministero della Salute’ (permit number: 6726-2011).

### Subjects

Sixty newborn guppies were used as subjects, 15 for each condition. Guppies are viviparous and give birth to fully developed offspring that are completely independent and display a full social repertoire [27]. To collect newborn guppies, adult females close to parturition were singly placed in nursery tanks. The sample size was preliminarily assessed by Fisher’s exact test [29]. A previous study in adult fish using the same methodology [30] showed that the standard deviation of the dependent variable is equal to 0.093, and the mean difference between two populations in a 0.50 discrimination is 0.115. As a consequence, by choosing α = 0.05 and a desired power of 0.80, even a sample of 11 individuals would be appropriate.

### Stimuli and Procedure

**Figure 1:**
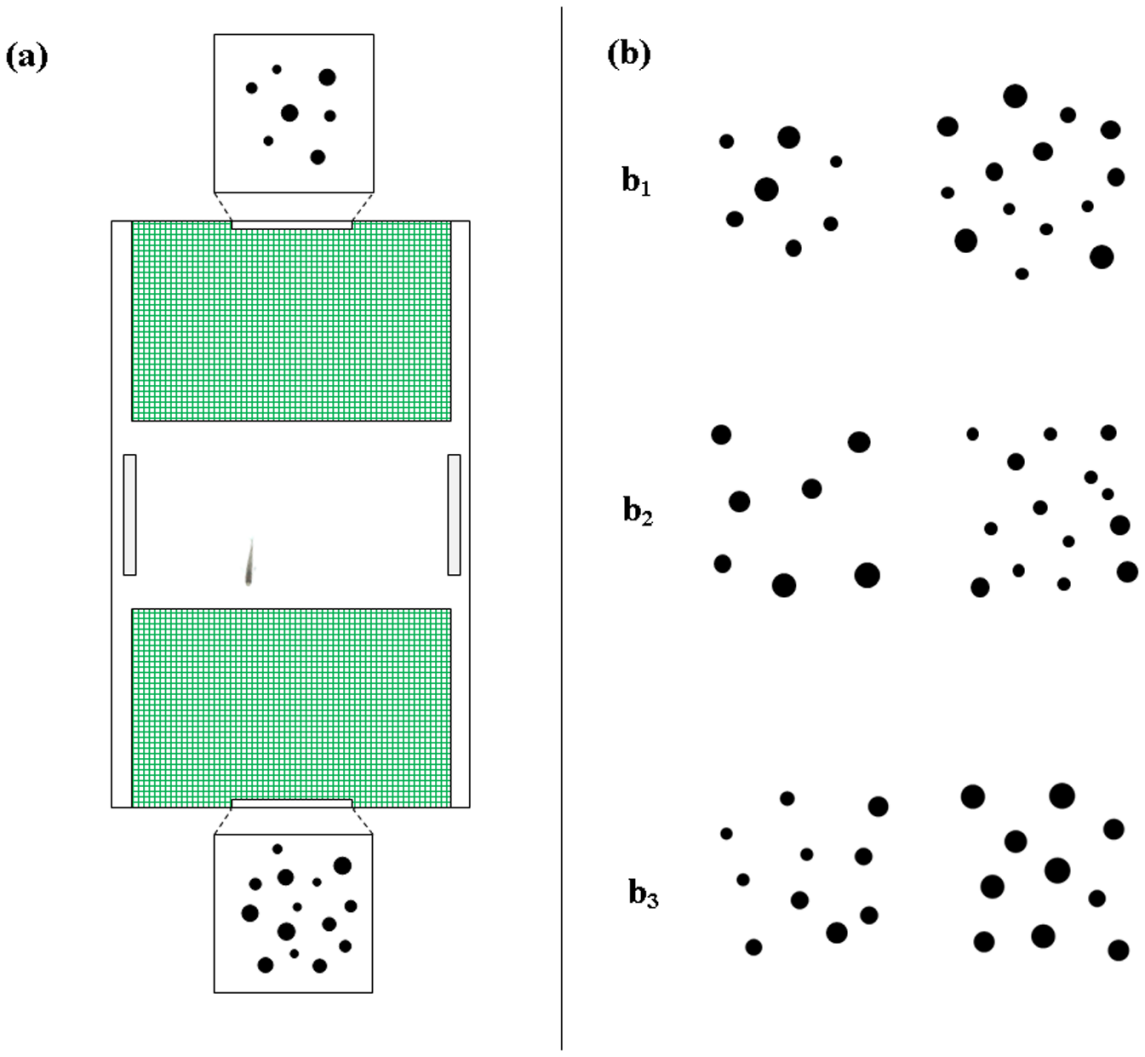
Experimental apparatus and stimuli. Newborn guppies were housed in an experimental tank for the entire experiment (a). Stimuli (groups of dots differing in numerosity and/or continuous quantities) were presented at the bottom of the tank. In the figure (b) we depicted a schematic representation of the stimuli used in the three experiments: number+continuous quantities (b1), only number (b2), only continuous quantities (b3).

We used the same apparatus and procedure previously utilized to investigate shape discrimination in newborn guppies [28]. The experimental apparatus (Fig. 1a) consisted of a 28 × 18.5 cm tank filled with gravel and 17 cm of water (25±2°C). At the two ends of the tank, corresponding to the sides at which the stimuli were presented, there were two tiles covered by a green net that delimited the preference areas (10×16 cm). The potential stress of social isolation was reduced by the presence of two mirrors (6×12 cm) placed in front of each other in the centre of the apparatus [28].

At birth, the fish were raised in groups, and after two days they were divided into groups of 5 and placed in the experimental apparatus. After 15 hours of habituation to the apparatus, the training phase began. Subjects were initially trained in groups of 5 from the 4^th^ to 5^th^ days of age (4 trials each day). Subsequently, from the 6^th^ to 8^th^ days of age, the fish were inserted in single apparatuses and trained individually. At the end of each daily session (days 6–8), the subjects were moved from one apparatus to another in order to avoid potential bias from the use of local or spatial cues of their tank.

In each trial, the subjects were presented with a pair of stimuli for 12 minutes. The stimuli were introduced at the two opposite ends of the apparatus and immediately followed by the reinforcement. A drop of live brine shrimps was used as positive reinforcement. It was released next to the correct stimulus, while as a control, a drop of water was released next to the other. The reinforcement was repeated after 6 minutes. As previous studies [21], [30], [31], [32] showed no influence of the direction of training (toward the larger or the smaller quantity as positive), all the subjects were trained to choose the largest (in term of number and/or continuous quantities, see below) stimulus of the pair. The inter-trial interval lasted 3 hours. The position of the reinforced quantity (left/right) was counterbalanced among trials. After five days of training (9 days of age), each subject singly underwent a probe trial during which stimuli were presented for 6 minutes and reinforcement not provided. Subsequently, three further training trials were given. On day 7, the subjects underwent a second probe trial, in which the position of the stimuli was reversed. In half probe trials, the reinforced quantity was presented on the right side, in the other half on left side. Stimuli used in probe trials were never shown during the training phase. The subjects’ performance was video-recorded during probe trials. For dependent variable in the two probe trials, we measured the proportion of time spent in the preference area (accuracy) around the reinforced stimulus.

The stimuli were pairs of groups of black dots on a white background (6×29 cm). Dots were distributed in a 5.5×5.5 cm central area and their size was different within the same pattern. They were subdivided into three different experiments.

#### Experiment 1: Number+continuous quantities

Both numerical and continuous quantities were available. Stimuli (diameter 0.29–0.65 cm) consisted of 7 and 14 dots (0.50 ratio), and, on average, the cumulative surface area of the largest group was double that of the smaller group. In addition, the brightness, density and overall space occupied by the groups were congruent with their numerosity (Fig. 1b).

#### Experiment 2: Only number

The contrast was 7 vs. 14, and the stimuli (diameter 0.29–0.65 cm) had the same continuous quantities: cumulative area, brightness, density and overall space. These quantities are known to be the only visual cues sometimes used by adult fish in their quantity judgements in presence of static stimuli [21], [31]. However, as a by-product of controlling for the cumulative surface area, the smallest dots were sometimes displayed systematically in the more numerous group, and the fish could have used this non-numerical cue (the size of the dots) instead of number. Consequently, in one-third of the pairs presented in the training phase, the cumulative area was matched to 100%, in one-third to 85%, and in the remaining third to 70%. Furthermore the biggest dot within each pair was shown half of the trials in the larger and half in the smaller set. The same rule was adopted for the smallest dot. Since overall space and density are inversely correlated half of the stimuli were controlled for space and half for density (Fig. 1b). This procedure was already adopted in a previous study investigating numerical abilities in five different fish species [33].

In probe trials we presented stimuli with identical features, with the exception that cumulative surface area was always matched to 100%.

#### Experiment 3: Only continuous quantities

The numerical information was made irrelevant, and only continuous information was available. The ratio between cumulative area of each pair was 0.50 (Fig. 1b). This condition was examined using two different numerical contrasts - 10 vs. 10 (diameter 0.29–0.65 cm) and 1 vs. 1 (diameter 1–1.88 cm) - following a recent work that suggested that the representation of continuous quantities in an array of multiple items might be more difficult to perceive than the representation of a single item [34].

Two tailed t-tests were performed to assess whether there were left-right biases and whether fish performance differed between the two probe trials; one tailed t-tests were performed to assess the a priori prediction [35], [36] according to which trained animals should spend more time near the reinforced stimulus. Data were arcsine (square root)-transformed [37]. The mean ± SD are provided. Statistical tests were carried out using SPSS 18.0.

## Results

Two subjects, one in the only number condition and one in the only continuous quantities (1 vs. 1) condition, were discarded at the end of the training phase due to poor health and excluded from the experiment.

### Experiment 1: Number+Continuous Quantities

Data had a normal distribution (Kolmogorov–Smirnov one sample t-test: 1^st^ probe trial, p = 0.998; 2^nd^ probe trial, p = 0.877). Fish accuracy did not differ between the 1^st^ (mean ± standard deviation: 0.58±0.23) and 2^nd^ probe trial (0.62±0.32; paired t-test: t_(14)_ = 0.704, p = 0.493). As a consequence in the following analyses we averaged the accuracy in the two probe trials.

**Figure 2:**
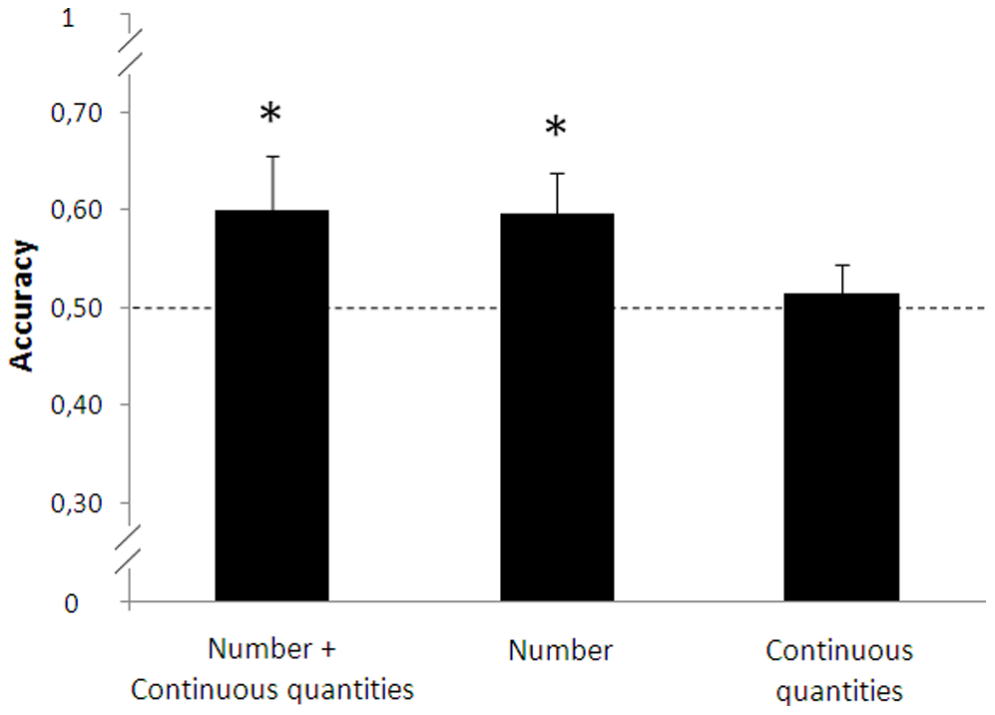
Results. Accuracy is plotted against the three experiments. Subjects proved themselves able to discriminate between 7 vs. 14 both when number and continuous quantities were available simultaneously and when only numerical information was used. The ability to discriminate between continuous quantities was not found. Asterisks denote a significant departure from chance level (p<0.05). Bars represent the standard error.

The position of the stimuli (left/right) did not affect the fish’s choice (left: 0.53±0.26, right: 0.68±0.28; paired t-test: t_(14)_ = 1.856, p = 0.085). The subjects successfully discriminated between the two quantities (one sample t-test: t_(14)_ = 1.829, p = 0.045, Fig. 2).

### Experiment 2: *Only Number*

Data had a normal distribution (Kolmogorov–Smirnov one sample t-test: 1^st^ probe trial, p = 0.788; 2^nd^ probe trial, p = 0.396). As fish accuracy did not differ between the 1^st^ (0.59±0.23) and 2^nd^ probe trial (0.59±0.24; paired t-test: t_(13)_ = 0.085, p = 0.934), we averaged the accuracy in the two probe trials.

The position of the stimuli (left/right) did not affect the fish’s choice (left: 0.58±0.22, right: 0.60±0.25; paired t-test: t_(13)_ = 0.338, p = 0.741). The subjects successfully discriminated between the two numbers (one sample t-test, t_(13)_ = 2.216, p = 0.023, Fig. 2). We found no difference in accuracy between trials controlled for density (0.58±0.25) and those controlled for overall space (0.60±0.22; t_(26)_ = 0.302, p = 0.951).

### Experiment 3: Only Continuous Quantities

In the 10 vs. 10 numerical contrast data had a normal distribution (Kolmogorov–Smirnov one sample t-test: 1^st^ probe trial, p = 0.959; 2^nd^ probe trial, p = 0.350). As fish accuracy did not differ between the 1^st^ (0.56±0.21) and 2^nd^ probe trial (0.50±0.26; paired t-test: t_(14)_ = 0.659, p = 0.520), we averaged the accuracy in the two probe trials.

Data had a normal distribution also in the 1 vs. 1 numerical contrast (Kolmogorov–Smirnov one sample t-test: 1^st^ probe trial, p = 0.560; 2^nd^ probe trial, p = 0.894). As fish accuracy did not differ between the 1^st^ (0.49±0.24) and 2^nd^ probe trial (0.46±0.22; paired t-test: t_(13)_ = 0.705, p = 0.493), we averaged the accuracy in the two probe trials.

The fish did not show any preference for the larger stimulus in either 10 vs. 10 (0.54±0.18) or 1 vs. 1 (0.49±0.14) contrast (one sample t-test, respectively: t_(14)_ = 0.843, p = 0.207; t_(13)_ = 0.348, p = 0.367). No difference between the two contrasts was observed (independent t-test: t_(27)_ = 0.870, p = 0.392). Also when the two samples were merged together in order to have a larger sample size (N = 29) fish did not show any preference for the larger stimulus (one sample t-test, t_(28)_ = 0.478, p = 0.636, Fig. 2). The position of the stimuli (left/right) did not affect the fish’s choice (left: 0.52±0.25, right: 0.49±0.21; paired t-test: t_(28)_ = 0.416, p = 0.680).

## Discussion

A previous study showed that at birth guppies cannot discriminate between shoals exceeding 4 units [27]. This finding has been interpreted as a complete absence of cognitive systems capable of performing large quantity discrimination until 20 to 40 days of age. However, other possibilities had not been excluded: that the large quantity system is present but imprecise at birth or that this system could be triggered by other experimental procedures in the first days of life. The purpose of the present study was to investigate the effect of training on the onset of large quantity discrimination.

We provided evidence that the ability to discriminate among large quantities is present in the first days of life. Guppies were trained from 4 days of age to discriminate between 7 and 14 dots; at the 10^th^ day, after only 20 trials, the fish learned how to perform large quantity discrimination, well before Bisazza et al. [27] observed the ability in shoal-choice tests. The developmental dissociation in the timing of the onset of quantity ability suggests that this new training procedure can speed up the emergence of cognitive abilities. This finding also highlights the importance of experience in the development of numerical abilities. However, we cannot exclude the possibilities that numerical systems in newborn guppies are stimuli-dependent and that the different performance observed in our study and in Bisazza et al.’s [27] study might be due the nature of the stimuli (i.e., biologically vs. non-biologically relevant stimuli or moving vs. non moving stimuli). Further study is needed to shed light on the issue.

Furthermore, in this study, we dissociated the relative contributions of numerical and continuous information. In experiment 1, the fish learned to discriminate when both numerical and non-numerical information were available. This result was not unexpected, because the combination of numerical and continuous information is the most common condition in nature (i.e., the largest group of food items often occupies also the largest area). In addition, this conclusion is in line with previous literature suggesting that redundancy of information might facilitate quantity discrimination in both human and non-human animals [32], [38], [39], [40].

Newborn guppies also solved the task in experiment 2 in which only numerical information was available. As density and overall space cannot be simultaneously controlled for, one might argue that fish could have used one of these non numerical cues, instead of number. However, no difference in accuracy was found between stimuli controlled for space and those controlled for density, which is incompatible with the hypothesis according to which density or overall space play a key role. This result agrees with findings in developmental psychology regarding the essential role played by numerical information. Indeed, both newborns and infants are known to discriminate among different quantities when continuous quantities are controlled for [41], [42]. Along with recent research on domestic chicks [43], [44], this is one of the first studies showing such a precocious ability to distinguish large numbers in non-human animals.

In contrast, in experiment 3, the fish failed to discriminate by using only continuous quantities–the opposite of what the last resort hypothesis predicts about numerical information. For newborn guppies, continuous quantities are not less cognitively demanding than numerical information, quite the opposite. A previous study on infants reported a better performance when a unique element (1 vs. 1) was presented [34]. Accordingly, in our study, we presented two different conditions (10 vs. 10 and 1 vs. 1) to test whether guppies’ performance followed that of infants. Guppies’ accuracy did not differ between the two conditions, showing a full inability to discriminate also when a unique element was presented. Trained guppies’ ability to process numerical information and inability to process continuous quantities can be interpreted in the light of Cantlon and Brannon’s [14] findings that, in a quantitative task, number-experienced monkeys based their discrimination more frequently on number than on continuous variables. The authors suggested that training might have increased the salience of numerical information.

Of course it remains the possibility that continuous quantity estimation might be partially affected by the position of the fish within the tank. For instance, a set of large dots seen at a certain distance might appear indistinguishable from a set of smaller dots seen at a closer distance. However we believe this is an unlikely explanation for the lack of discrimination here observed, as a previous study in newborn guppies using the same apparatus and procedure [28] showed a successful discrimination between geometric figures having very similar size compared to most of the dots used in experiment 3.

The results of experiment 2 and 3 have potential implications for the debate surrounding the cognitive mechanisms processing number and space. It was suggested [45] that non-symbolic number estimation of humans is processed by the same cognitive mechanism involved for other magnitudes, the so-called ‘a theory of magnitude’ (ATOM). In short, the same mechanism would be recruited when people estimate which tone lasts longer (time), which area is larger (space), and which group of dots is more numerous (numbers). Both behavioral [46], [47] and neuroimaging [48] studies support this view. Here we found that number acuity is better than spatial acuity in newborns, while adult fish are known to discriminate both numerical and spatial information apparently with the same cognitive effort [32]: the different ontogeny of numerical and spatial abilities points toward the existence of separate cognitive systems for these magnitudes. Even though we can only speculate on this topic, it is possible that the common magnitude system advanced by Walsh [45] is a recent evolutionary development restricted to humans or, more generally, to closely related species to humans.

It is worth remembering that our method differs from a standard operant conditioning procedure. The fact that discrimination is inferred from the proportion of time spent near the trained numerosity during final probe trials limits the possibility to obtain detailed information about the learning process (as commonly reported using standard procedures). Nonetheless, reaching a learning criterion in operant conditioning procedures usually requires hundreds or thousands of trials [31], [49], [50], which is clearly ineffectual if we need to study cognitive skills in a fast growing species, such as guppies.

As a last note, the difference here observed between free choice tests [27] and the training procedure in our study highlights a widespread problem of the literature, namely the difficulty to draw any firm conclusion when single studies are not replicated. Replication in animal cognition should be encouraged not only by using the same methodology (to assess whether initial results are simply an accident) but also by using different methods and approaches, to determine whether previous results are universal or context-dependent (i.e., ecological context, procedure, stimuli, expertise, etc.). If different methods lead to different conclusions [51], [52], the next decade is expected to be characterized by an increased attention in replication studies.
